# Fc*γ* and Complement Receptors and Complement Proteins in Neutrophil Activation in Rheumatoid Arthritis: Contribution to Pathogenesis and Progression and Modulation by Natural Products

**DOI:** 10.1155/2015/429878

**Published:** 2015-08-05

**Authors:** Adriana Balbina Paoliello-Paschoalato, Larissa Fávaro Marchi, Micássio Fernandes de Andrade, Luciana Mariko Kabeya, Eduardo Antônio Donadi, Yara Maria Lucisano-Valim

**Affiliations:** ^1^Department of Physics and Chemistry, School of Pharmaceutical Sciences of Ribeirão Preto, University of São Paulo, Avenida Café s/n, 14040-903 Ribeirão Preto, SP, Brazil; ^2^Department of Internal Medicine, Ribeirão Preto Medical School, University of São Paulo, Avenida Bandeirantes 3900, 14049-900 Ribeirão Preto, SP, Brazil; ^3^Department of Biochemistry and Immunology, Ribeirão Preto Medical School, University of São Paulo, Avenida Bandeirantes 3900, 14049-900 Ribeirão Preto, SP, Brazil

## Abstract

Rheumatoid arthritis (RA) is a highly disabling disease that affects all structures of the joint and significantly impacts on morbidity and mortality in RA patients. RA is characterized by persistent inflammation of the synovial membrane lining the joint associated with infiltration of immune cells. Eighty to 90% of the leukocytes infiltrating the synovia are neutrophils. The specific role that neutrophils play in the onset of RA is not clear, but recent studies have evidenced that they have an important participation in joint damage and disease progression through the release of proteolytic enzymes, reactive oxygen species (ROS), cytokines, and neutrophil extracellular traps, in particular during frustrated phagocytosis of immune complexes (ICs). In addition, the local and systemic activation of the complement system contributes to the pathogenesis of RA and other IC-mediated diseases. This review discusses (i) the participation of Fc*γ* and complement receptors in mediating the effector functions of neutrophils in RA; (ii) the contribution of the complement system and ROS-dependent and ROS-independent mechanisms to joint damage in RA; and (iii) the use of plant extracts, dietary compounds, and isolated natural compounds in the treatment of RA, focusing on modulation of the effector functions of neutrophils and the complement system activity and/or activation.

## 1. Introduction

Rheumatoid arthritis (RA) occurs in 0.5–1.0% of the adult population worldwide and accounts for around 250,000 hospitalizations and 9 million doctor visits per year [1]. Twenty to 30% of the untreated RA patients become unable to work within three years of diagnosis [2]. RA is a chronic inflammatory polyarthritis disease that affects multiple joints, and some types of RA also affect multiple organ systems. RA is characterized by synovial hyperplasia, swelling, pain, and neutrophil-rich infiltrates and can lead to bone erosion, cartilage destruction, and complete loss of joint integrity over time. This condition is classified as an autoimmune disorder because it involves the formation of antibodies against self-antigens causing immune complex (IC) deposits in synovial tissue of patients with RA [3, 4].

RA is a multifactorial disease in which genetic, environmental, and immunologic factors contribute to disease outcome and progression [5]. Studies have confirmed the key role of the major histocompatibility complex genes and identified other loci that warrant further exploration [6]. The prevalence of RA in various populations has been associated with increased urbanization and other factors like cigarette smoking [6–8]. Smokers usually exhibit augmented concentrations of rheumatoid factors and anti-cyclic citrullinated peptide (anti-CCP) antibodies, as well as disturbances of immune functions and redox balance [5]. Autoantibodies are one immunologic factor that significantly participates in the etiology of RA. The rheumatoid factors—which are autoantibodies directed to the Fc fraction of immunoglobulin G (IgG)—and anti-CCP antibodies can be detected in the preclinical phase of the disease. The levels of these antibodies tend to increase as a function of the age at diagnosis of RA [9]. Around 10–50% of RA patients have anti-collagen II antibodies, and some patients with very severe arthritis have anti-glucose-6-phosphoisomerase antibodies [10].

The disease progression and the therapeutic efficacy of RA treatment can be monitored using the disease activity score of 28 joints (DAS-28), which is calculated from (i) the number of painful joints (hands, arms, and knees); (ii) the number of swollen joints (hands, arms, and knees); (iii) the visual analogue scale of patients' assessment of their general health; (iv) the erythrocyte sedimentation rate in the first hour and/or the blood level of C-reactive protein to measure the degree of inflammation. The DAS-28 score correlates with the extent of disease activity as follows: <2.6: disease remission; >2.6 and <3.2: low disease activity; >3.2 and <5.1: moderate disease activity; >5.1: high disease activity [11]. Other laboratory tests used to diagnose RA and follow disease progression include total and differential blood cell count, evaluation of renal and hepatic function, urinalysis, and measurement of plasma levels of complement, antinuclear antibody, anti-CCP antibody, and immunoglobulins [12].

The pathogenesis of RA remains unclear but it is known that the cellular and humoral components of the immune system are activated and they coordinately contribute to disease pathology (see [13] for review). CD4^+^ T cells, B cells, macrophages, and neutrophils are present in synovial infiltrate, and these cells sometimes organize into discrete lymphoid aggregates with germinal centers [4]. During the active phases of RA, 80 to 90% of the immune cells infiltrating the synovial fluid (SF) are neutrophils; the neutrophil turnover can exceed 10^9^ cells per day in a 30 mL joint effusion [13–15]. Neutrophil production in the bone marrow is augmented in RA patients, and both mature and immature neutrophils are mobilized [16, 17]. The neutrophil-lymphocyte ratio and platelet-lymphocyte ratio are markers of systemic inflammation that correlate with DAS-28 scores in patients with RA [18].

In acute and chronic inflammatory responses, neutrophils communicate with other innate and adaptive immune system cells through direct cell-cell contact and the release of neutrophil extracellular traps (NETs), cytokines, granule components, reactive oxygen species (ROS), and other soluble mediators. The complex cross talk between neutrophils and immune cells is essential to drive and control the course of inflammatory and autoimmune diseases [19, 20]. Many researchers have recently reviewed the novel immunomodulatory functions of neutrophils [19–21].

Neutrophils are important players in promoting systemic and local (in the synovia) oxidative stress in patients with RA [22–24]. The oxidant status of neutrophils usually correlates with DAS-28 scores and the level of oxidative stress markers and tissue damage in patients with RA [25, 26]. Deposition of ICs and complement proteins onto the joint surface impairs the complete phagosome closure, resulting in frustrated phagocytosis. Neutrophils then release high amounts of oxidant and cytotoxic agents into the microenvironment of the semiclosed phagosome, that is, the joint surface and SF. Massive neutrophil infiltration and activation can overwhelm the local antiprotease and antioxidant protective mechanisms and damage the surrounding tissues of patients with RA [19, 22–24, 27, 28].

Given the importance of ICs deposited in the RA patients' synovia to elicit the effector functions of neutrophils via Fc*γ* and complement receptors (Fc*γ*R and CR, resp.), the present paper aims to discuss the participation of these receptors and complement proteins in the production of ROS and release of granule components by neutrophils in the inflamed synovia and in peripheral blood. This paper also discusses the future perspectives in the treatment of RA patients with plant extracts, dietary compounds, and isolated natural compounds to minimize the harmful effects of the overactivation of neutrophils and the complement system.

## 2. Fc*γ*Rs and CRs and the Complement System in Neutrophil Activation: Contribution to Pathogenesis and Progression of RA

Neutrophils express many types of cell surface receptors, including Fc*γ*R, CR, G-protein-coupled chemokine and chemoattractant receptors, adhesion receptors, Toll-like receptors, and C-type lectins. Some types of receptors are not constitutive, but their expression can be induced by molecules of the inflammatory environment (see [29] for review).

**Figure 1:**
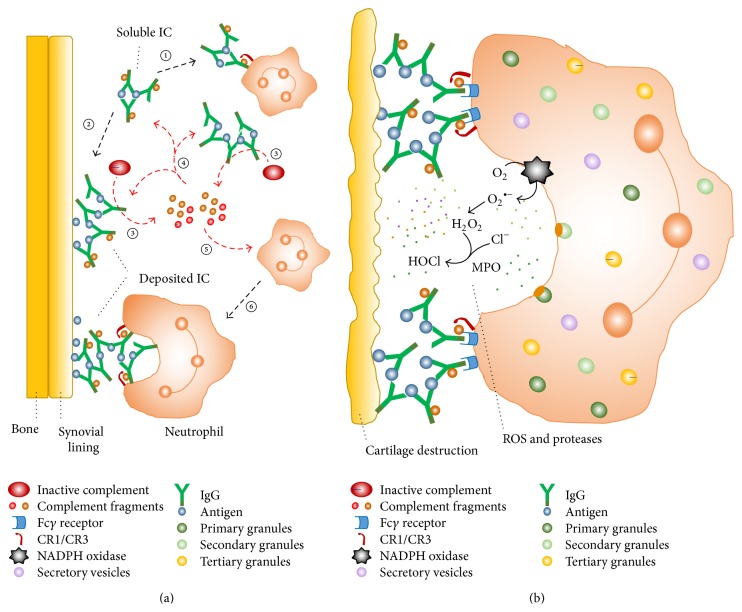
Interaction among neutrophils, immune complexes, and the complement system in mediating joint damage in rheumatoid arthritis. (a) Soluble ICs in the rheumatoid synovial fluid can bind and stimulate primed neutrophils (①) or deposit in the synovial lining (②). Both soluble and deposited ICs activate the complement system (③), generating protein fragments; these components of the activated complement system deposit in synovial tissues, opsonize soluble and tissue-bound ICs (④), and attract neutrophils to the inflamed joint (⑤). The recruited neutrophils recognize IgG and some complement fragments contained in the ICs via their Fc*γ* and complement receptors, respectively (⑥). (b) Tissue-bound ICs activate neutrophils, which are not able to phagocytose them; as a consequence, neutrophils release ROS and proteolytic enzymes from their granules and secretory vesicles to the extracellular milieu. This process is termed “frustrated phagocytosis.” The cytotoxic products can overwhelm the local antiprotease and antioxidant protective mechanisms and degrade components of articular cartilage. CR1/CR3, complement receptors types 1 and 3; IC, immune complex; IgG, immunoglobulin G; MPO, myeloperoxidase; ROS, reactive oxygen species. This illustration was adapted from the review article published by Wright et al [21], with permission of Macmillan Publishers Limited.

In the inflamed synovia, the neutrophil surface Fc*γ*Rs and CRs cooperate to recognize ICs opsonized with complement system proteins and further activate the cellular effector functions. Both soluble ICs and insoluble ICs deposited along the synovial membrane lining are recognized by cytokine-primed neutrophils in the inflammatory microenvironment. Therefore, the interaction among ICs, proteins of the complement system, and Fc*γ*Rs and CRs to activate the oxygen-dependent and oxygen-independent functional responses of neutrophils markedly contributes to the pathogenesis and progression of RA [13, 21, 30, 31]. This scenario is depicted in Figure 1.

This paper will mainly discuss how the complement system and neutrophil Fc*γ*Rs and CRs participate in the pathogenesis and progression of human RA. The role that these components of the immune system play in animal models of arthritis has been reviewed elsewhere [32, 33].

### 2.1. Fc*γ*Rs

Neutrophil-mediated tissue damage triggered by ICs is the hallmark of many inflammatory diseases. ICs present in the SF, in the superficial layers of the cartilage, and circulating in the periphery interact primarily with Fc*γ*Rs expressed on the neutrophil surface [31, 34]. The levels of circulating ICs correlate positively with DAS-28 scores in RA patients [35]. Priming neutrophils with cytokines regulates their functional responses to these ICs. Soluble ICs that exist in the SF elicit a rapid, intense, and transient ROS generation in GM-CSF-primed neutrophils, but not in unprimed neutrophils. Resting neutrophils are efficiently stimulated* in vitro* by large immunoglobulin aggregates and insoluble ICs [31, 36].

The Fc*γ*Rs and CRs are engaged in the clearance of ICs, antigen presentation and uptake, antibody-dependent cellular cytotoxicity, and the neutrophil-mediated damage within the synovial joint [37]. Neutrophil activation via Fc*γ*R also elicits degranulation, production and release of cytokines, expression of cyclooxygenases and nitric oxide synthase, and synthesis of leukotrienes and arachidonic acid metabolites [30, 38, 39]. Fc*γ*Rs cooperate with other families of receptors in controlling local and systemic cytokine production, which is essential to shape both innate and adaptive immune responses in infectious, inflammatory, and autoimmune diseases (see [40] for review).

Fc*γ*R classes differ in relation to affinity for IgG, tissue distribution, and expression level. Human neutrophils constitutively express the low-affinity Fc*γ*RIIa (CD32a) and Fc*γ*RIIIb (CD16b) receptors. Activation of human neutrophils by ICs requires synergistic ligation of both activating receptors [29]. Fc*γ*RIIa expression in neutrophils is upregulated by priming agents such as tumor necrosis factor-*α* (TNF-*α*) and granulocyte-macrophage colony-stimulating factor (GM-CSF) [41]. Peripheral blood neutrophils from RA patients with active disease express elevated levels of Fc*γ*RIIa, as compared with neutrophils from RA patients with inactive disease and healthy individuals [42]. The TNF-*α* inhibitor infliximab reduces the expression of the activating Fc*γ*RIIa in RA patients' neutrophils* in vivo* [41].

Fc*γ*RIIIb is highly abundant in and exclusively expressed on neutrophils. This receptor is constantly shed from the surface of activated neutrophils by metalloproteases and replaced by new receptors stored in intracellular granules [31]. Binding of soluble ICs to Fc*γ*RIIIb predominantly elicits the extracellular secretion of ROS and proteases, while the recognition of insoluble ICs by Fc*γ*RIIa predominantly triggers intracellular ROS production in neutrophils [43]. In a murine model of antibody-mediated arthritis, only neutrophils that expressed the activating Fc*γ*RIIa and Fc*γ*RIIIb receptors were able to migrate to synovial tissues containing deposited ICs [44].

The neutrophil Fc*γ*RIII is the dominant activating receptor that mediates joint inflammation, whereas both Fc*γ*RI and Fc*γ*RIII mediate cartilage destruction [45]. Fc*γ*RIII expression on monocytes and the proportion of Fc*γ*RIII-positive monocytes are also increased in RA patients with active disease [46]. Fc*γ*RIIIa gene polymorphism is associated with increased susceptibility to RA [47] and influences treatment outcomes in patients with RA or psoriatic arthritis treated with TNF-*α* blocking drugs and rituximab [48, 49]. Polymorphisms in the activating Fc*γ*RIIc receptor, expressed only in a minority of individuals, are associated with disease severity in RA patients [50].

Activated but not resting blood neutrophils express Fc*γ*RI (CD64), the high-affinity receptor for monomeric IgG [29]. Fc*γ*RI expression can be induced by treating healthy blood neutrophils with IFN-*γ* and cell-free RA patients' SF. RA patients' SF neutrophils that express Fc*γ*RI exhibit increased ability to respond to IgG-containing ICs present in the joint [51].

Human neutrophils express the inhibitory Fc*γ*RIIb receptor that negatively regulates cell activation. Fc*γ*RIIb inhibits joint inflammation and cartilage destruction in IC-mediated arthritis [45]. Defective Fc*γ*RIIb function is associated with increased disease severity and joint erosions in RA patients [52].

### 2.2. The Complement System

The complement system is an essential component of the innate immune system that participates in microbial killing, clearance of apoptotic cells and ICs, recruitment of inflammatory cells, and regulation of immune responses and inflammatory processes [30, 53, 54]. In addition, the complement system constitutes a network connected to other systems such as the kallikrein-kinin system and the blood coagulation system [53, 55].

The complement cascade reaction can be initiated by three major pathways: classical, alternative, and lectin pathways. The classical pathway is activated when C1q recognizes the Fc portion of ICs and clustered IgG and/or IgM antibodies bound to their targets. The alternative pathway is initiated by cleavage of the unstable complement factor C3—that can occur spontaneously or after its interaction with pathogen's cell surfaces—and subsequent deposition of C3b on microbial cell surfaces [10, 53]. Under certain circumstances, the alternative pathway seems to be activated by properdin and ICs composed of IgG and IgA [56]. The lectin pathway is triggered by the binding of microbial polysaccharides to circulating lectins, such as plasma mannan-binding lectins or ficolins, structurally similar to C1q. The alternative and lectin pathways are activated in the absence of antibodies [10, 53].

The three pathways of the complement system activation have overlapping reactions and can be activated simultaneously* in vivo* [53, 55]. These pathways of complement activation lead to cleavage of the complement proteins C2, C3, C4, and C5 as well as to production of the anaphylatoxins C3a and C5a and the opsonins C3b, C3d, and iC3b. They also culminate in the assembly of the complement components C5b, C6, C7, and C8 and multiple copies of C9 to form the C5b-9_(*n*)_ complex called “terminal complement complex” or “membrane attack complex” (MAC). When assembly occurs on a target cell membrane, MAC inserts into and through the bilayer to create a pore and induce cell lysis [53, 57].

The complement system is composed of approximately 50 components, including soluble and cell-bound complement proteins, convertases, and regulatory proteins, which act together in immune regulation [58]. The complement system participates in the pathogenesis of human IC-mediated diseases, such as systemic lupus erythematosus (SLE), vasculitis, glomerulonephritis, and RA (see [53–55] for review). In RA, the complement system is activated by circulating and deposited ICs, rheumatoid factors, anti-CCP antibodies, IgG molecules with altered glycosylation patterns, C-reactive protein, and surface molecules exposed after cartilage damage [54, 59].

The local production and deposition of complement components in RA patients' synovial tissues was first demonstrated in 1974 [60]. The rheumatoid synovia of patients with RA and osteoarthritis usually exhibits reduced levels of complement proteins C3, C4, and factor B, associated with increased levels of complement metabolites and the soluble form of MAC (sMAC or sC5b-9_(*n*)_) [61]. Synovial complement activation positively correlates with the intensity of joint inflammation and disease activity in patients with RA [32, 55].

The C5a complement fragment is a potent neutrophil chemoattractant and priming agent. In the RA patients' SF, the C5a level is increased and correlates positively with the number of infiltrating neutrophils [60, 62]. C5a also amplifies the local joint inflammation by directly activating neutrophils via C5a receptor (C5aR, CD88) and by increasing complement receptor 3 (CR3) activity, which in turn upregulates neutrophil accumulation [20, 63]. The levels of C3a receptor (C3aR) and C5aR expression in rheumatoid synovia are augmented [61]. C3a and C5a seem to mediate the activation of NLRP3 inflammasome signaling pathway, which has an important participation in RA inflammatory processes; however, the mechanisms by which these complement fragments act were not fully elucidated [57].

Interestingly, RA patients with active disease exhibit elevated C5a and Bb serum levels and increased activity of the alternative complement pathway. Serum obtained from RA patients with active disease induces neutrophil chemotaxis* in vitro* [42]. The plasma levels of the C1q-C4 complex, produced during the early stage of the classical pathway activation, are higher in RA patients with active disease, as compared with RA patients in clinical remission [64]. The plasma levels of C3 correlate positively with the plasma levels of C4, C3d, and circulating ICs and DAS-28 scores in RA patients with active disease [35]. These findings demonstrate that systemic activation of the complement system plays an important role in clinical manifestations of RA.

Extensive deposition of MAC induces cell lysis and necrotic cell death, but sublytic amounts of MAC play a variety of roles in inflammatory processes (see [57] for review). Although MAC removal from the plasma membrane either by ectocytosis or by endocytosis allows cells to survive and recover, exposure to sublytic amounts of MAC alters the cell physiology [57]. Sublytic concentrations of MAC activate granulocytes, endothelial cells, and platelets, induce apoptosis, elicit degranulation and synthesis of cytokines in neutrophils and macrophages, and affect intracellular signaling by interacting with membrane receptors and increasing both Ca^2+^ influx and release from intracellular stores [55, 57]. These proinflammatory effects of nonlethal concentrations of MAC contribute to amplify joint inflammation in RA.

Decreased tissue and SF levels of the MAC inhibitors clusterin, vitronectin, and CD59 are associated with lytic or sublytic attacks on synovial cells [55]. The elevated plasma levels of sMAC correlate negatively with CD59 expression levels in patients with psoriatic arthritis in the active phase of the disease [65]. In cartilage from patients with end-stage osteoarthritis, MAC is present in the synovium and around the chondrocytes, and the expression of mRNA encoding the complement components C7, C4a, factor B, C9, and C5 is augmented, while the expression of mRNA encoding the complement inhibitors clusterin, factor H, C4-binding protein, and C1 inhibitor (Serping1) is diminished [66].

Patients with RA and osteoarthritis exhibit increased plasma and SF levels of sMAC, which occur as a mixture of inactive sMAC and fluid-phase sMAC [67]. RA patients with active disease also have elevated plasma and SF levels of osteoprotegerin, which is a soluble member of the TNF receptor superfamily that suppresses osteoclast formation [68]. sMAC upregulates the production and expression of osteoprotegerin in endothelial cells, and both molecules induce the adhesion of leukocytes to endothelial cells. Hence, sMAC and osteoprotegerin may promote leukocyte extravasation in RA patients' synovia and thereby contribute to promote and/or enhance joint inflammation [68].

MAC binding to synovial fibroblasts stimulates them to produce collagenase and ROS and to proliferate, resulting in the joint damage and synovial hypertrophy characteristic of RA [69–71]. The activated synovial fibroblasts also act as effector cells in joint inflammation through the production of leukocyte chemoattractants, matrix metalloproteinases (MMPs), proinflammatory mediators, and growth factors [70]. In chondrocytes isolated from human osteoarthritic cartilage, sublytic amounts of MAC increase the expression of genes encoding cartilage-degrading enzymes like MMPs and metallopeptidases, proinflammatory cytokines and chemokines, and the complement effectors C3, C5, and factor B [66]. Products of dysregulated cartilage remodeling and repair induce the formation of MAC* in vitro*; thus, they can contribute to joint inflammation [66].

Sublytic concentrations of MAC induce citrullination of intracellular proteins of different molecular weights, a process termed “cellular hypercitrullination,” in trinitrobenzene sulfonate-treated SF neutrophils and monocytes from RA patients [72]. Citrullination—a posttranslational modification catalyzed by peptidylarginine deiminases (PADs), where peptidyl-arginine residues are converted to citrulline—markedly contributes to generate the profile of citrullinated autoantigens characteristic of RA [73]. In RA patients' neutrophils, hypercitrullination seems not to be linked to apoptotic cell death but is associated with NETosis. Neutrophils and monocytes are the major sources of PADs in the synovia [72, 74].

### 2.3. CRs

Phagocytic cells bear membrane receptors that recognize soluble and antigen-bound complement components. Neutrophils constitutively express CR1 (CD35), CR3 (CD11b/CD18, Mac-1), and CR4 receptors. CR1 binds C1q, C4b, C3b, iC3b, C3b/C4b complexes, and the mannan-binding lectin and cooperates with other receptors to trigger the functional responses of neutrophils. CR3 preferentially binds C3bi and elicits phagocytosis, degranulation, ROS generation, and neutrophil migration [53, 56, 75, 76]. CR3 engagement, clustering, and activation are involved in the signaling that drives neutrophil apoptosis [77].

Mobilization of intracellular granules during neutrophil activation increases the expression of CR1 on the neutrophil surface [20]. Peripheral blood neutrophils and monocytes from RA patients with active disease express increased levels of CR1 and CR3 receptors, when compared with leukocytes from RA patients with inactive disease and healthy individuals [42, 46]. The expression levels of both receptors in SF neutrophils were higher than the expression levels in peripheral blood neutrophils and correlated with the number of neutrophils in the RA patients' SF [78, 79]. Another study reported that, compared with healthy individuals' leukocytes, RA patients' peripheral blood leukocytes exhibit lower expression levels of CR1 mRNA, which negatively correlates with DAS-28 score and the levels of circulating ICs and C3d [35]. CR1 is an important complement regulatory protein that has been considered as a potential disease marker for RA [35].

On the other hand, CR1 and CR2 expression levels on RA patients' B cells are diminished [80]. RA patients also exhibit decreased serum levels of soluble CR2 receptor (sCD21). sCD21, the extracellular portion of the CD21 glycoprotein released by shedding from cell surfaces into plasma, binds degradation fragments of iC3b, such as C3dg and C3d, and may activate monocytes [81].

### 2.4. Interaction between Fc*γ*Rs and CRs in the Neutrophil Activation in RA

RA patients' blood neutrophils are functionally very different from healthy individuals' blood neutrophils: the former are primed for ROS production whilst the latter exist in a resting state in the circulation. These types of neutrophils also differ in their gene and protein expression patterns [21]. To date, few studies have examined how the cooperation between Fc*γ*Rs and CRs contributes to the pathogenesis and progression of RA and other autoimmune diseases [32, 39, 42, 82].

The low-affinity Fc*γ*Rs act in synergy with CRs to mediate the safe removal of ICs and opsonized particles [30]. The simultaneous binding to CRs and Fc*γ*Rs may enhance the efficiency of phagocytosis by leukocytes. The neutrophil CR1, CR3, Fc*γ*RIIa, and Fc*γ*RIIIb receptors promote IC adherence and increase the efficiency of phagocytosis, oxidative burst, and degranulation, as compared with IC interaction with each receptor alone, providing evidence of synergy among these receptors [38, 39, 75].

In a study comprising RA patients stratified according to DAS-28 score, the Fc*γ*RIIa and CR1 expression levels in neutrophils from RA patients with active disease were higher than the expression levels of these receptors in neutrophils from healthy controls and RA patients with inactive disease [42]. Peripheral blood neutrophils from RA patients with active and inactive disease respond to IC opsonized with healthy individuals' sera (exogenous complement source) with significantly increased ROS production, when compared with nonopsonized IC. Hence, Fc*γ*Rs and CRs cooperate in eliciting ROS production in RA patients' peripheral blood neutrophils [42]. In the same study, ICs opsonized with autologous sera and nonopsonized ICs elicited equal levels of ROS production in RA patients' neutrophils. This finding provides an evidence that the RA serum-opsonizing capacity is diminished due to systemic activation of the complement system in RA patients [42].

The cooperation between C5aR and Fc*γ*Rs initiates and sustains neutrophil recruitment in an animal model of RA [83, 84]. C5aR-mediated activation of neutrophils is required for leukotriene B4 release and early neutrophil recruitment to the joint, whereas Fc*γ*R engagement in neutrophils induces interleukin- (IL-) 1*β* release, ensuring continued inflammation [83]. Simultaneous activation of neutrophil Fc*γ*RIIb and dectin-1 by highly galactosylated IgG downregulates the functional response of C5aR [85]. The anaphylatoxin C5a downregulates the inhibitory Fc*γ*RII receptor expression, which in turn upregulates the Fc*γ*RI and Fc*γ*RIII expression in activated neutrophils [32]. These data support the concept that IC-mediated leukocyte activation is not composed of overlapping and redundant pathways but that each element serves a distinct and critical function* in vivo*, culminating in tissue inflammation. The CRs also play significant roles in articular inflammation [55]. Mice lacking C5aR or C3aR have milder disease in arthritis models; it suggests that the production of anaphylatoxins and their interaction with cellular receptors contribute, at least in part, to the pathogenesis of RA [83, 84].

In a group of Brazilian SLE patients with active disease, the reduced expression of Fc*γ*RII and CR1 impaired their cooperation in mediating the neutrophil oxidative burst but did not affect the neutrophil degranulation capacity [39]. The clinical manifestations of the disease clearly correlate with the kinetics of O_2_^•−^ production and efficiency of Fc*γ*R/CR cooperation in SLE patients' neutrophils—these functions can be increased or decreased, depending on the levels of circulating ICs and cytotoxic antibodies [82].

## 3. ROS-Dependent and ROS-Independent Tissue Injury Mechanisms in RA

The release of granule components and the production of ROS by neutrophils are the main tissue injury mechanisms in RA. Some scholars argue that these processes are complementary while other researchers have attempted to determine which of these factors contributes more to the pathological processes of RA [19]. For didactic purposes, we have separated the ROS-dependent and ROS-independent mechanisms. In each topic, we discuss how these mechanisms interact to cause joint damage.

### 3.1. ROS-Independent Mechanisms

#### 3.1.1. Degranulation

Upon stimulation, neutrophils release proteolytic enzymes from their granules and secretory vesicles. Three types of granules are formed consecutively during neutrophil maturation: azurophilic or primary granules, specific or secondary granules, and gelatinase or tertiary granules. The secretory vesicles are the last granule type formed in neutrophils, but they are quickly mobilized to the cell surface during priming and activation; the proteins contained in such vesicles are incorporated into the plasma membrane [86]. In IC-mediated diseases such as RA, extracellular release of granule contents usually occurs due to frustrated phagocytosis. Increased degranulation and delayed apoptosis of neutrophils correlate with the intensity of synovial inflammation and the destructive capacity of joint neutrophils in RA [21, 24, 87]; however, changes in the physiology and function of peripheral blood neutrophils are less known.

The granules' components and ROS degrade polysaccharides and other high-mass components of articular cartilage to form low-mass products, such as acetate and formate. This reaction is facilitated by immunoglobulins bound to the cartilage surface and by the synergistic action of neutrophil serine proteases, metalloproteases, and ROS released during activation by surface-associated immunoglobulins [88, 89]. Human neutrophils, neutrophil granule extracts, and purified elastase degrade proteoglycans and inhibit proteoglycan synthesis in IgG-coated human and bovine cartilage [88]. Some researchers consider the enzyme elastase, usually found in primary granules, as the most relevant enzyme for cartilage damage. Specific elastase inhibitors like* N*-methoxysuccinyl-(ala)_2_-pro-val-chloromethylketone, but not protease inhibitors lacking antielastase activity, prevented and partially reversed cartilage degradation induced by neutrophils stimulated* in vitro* [88, 90].

Lactoferrin is found in specific or secondary granules of neutrophils. The lactoferrin concentration in the RA patients' SF and sera is significantly higher than in these fluids collected from patients with osteoarthritis. The lactoferrin concentration in healthy subjects' SF ranges from 2 to 7 *μ*g mL^−1^, but it can be as high as 100 *μ*g mL^−1^ in RA patients' SF [91]. Lactoferrin displays a regulatory function at the site of joint inflammation by regulating the expression of genes encoding bone morphogenetic proteins via the mitogen-activated protein kinase ERK pathway. The lactoferrin expression levels in the membrane of SF neutrophils are elevated in comparison with its expression levels in peripheral blood neutrophils of patients with RA and osteoarthritis [92]. Lactoferrin can delay neutrophil apoptosis and probably acts as a survival factor for SF neutrophils [93]. These results provide evidence for the activation of neutrophils at the site of inflammation in RA and indicate that lactoferrin surface expression represents a reliable neutrophil activation marker. However, the expression levels of lactoferrin and other cell activation markers at the site of inflammation in RA do not always correlate with the systemic disease activity [91–93].

#### 3.1.2. NETosis

Activated neutrophils can release NETs to the extracellular space, in a process termed NETosis. NETs are neutrophil nuclear DNA fibres associated with histones and granule components, such as elastase, myeloperoxidase, lactoferrin-chelating proteins, LL-37, and other antimicrobial molecules that disarm and kill bacteria extracellularly [94, 95]. NETosis, phagocytosis, degranulation, and ROS generation represent the main strategies that neutrophils employ to fight against pathogenic microorganisms [96].

Neutrophils release NETs via two central mechanisms (see [96, 97] for review). The major route is the slow (120–240 min) lytic cell death mechanism, also called “lytic NETosis” or “suicidal NETosis,” which is characterized by plasma membrane rupture to release NETs and cell death and requires the activation of NADPH oxidase and production of ROS [96, 97]. The other pathway, termed “vital NETosis” or “nonsuicidal NETosis,” involves the rapid (5–60 min) release of vesicles containing decondensed chromatin and granule proteins in the extracellular space, where they assemble into NETs—this process renders NETs and live intact cytoplasts without signs of cell death that continue to crawl slowly and digest microbes; therefore, it does not result in neutrophil lysis and death [97, 98].

In contrast to apoptosis, an anti-inflammatory mechanism of cell death that stimulates tissue repair processes, NETosis is a proinflammatory event [20]. It is not clear which factors drive the neutrophil's death via NETosis or apoptosis. It seems to be related to the type of stimulus, as well as to the intensity and duration of cell stimulation. There are some evidences that precipitated and soluble ICs preferentially stimulate apoptosis and NETosis, respectively [20, 21]. Antibodies and ICs induce NETosis less effectively than pathogenic bacteria, fungi, and HIV parasites. Activated platelets, lipopolysaccharides, cytokines, and chemical compounds like phorbol-12-myristate-13-acetate (PMA) also trigger NETosis in primed neutrophils [95]. The proinflammatory cytokines IL-17a and TNF-*α*, whose levels are elevated in RA patients' sera, do not trigger NETosis in naïve neutrophils but elicit NETosis in RA patients' neutrophils not primed by ROS. Anti-IL-17a and anti-TNF-*α* antibodies mitigate the NET-inducing ability of RA patients' sera [74].

As deficiencies that impair NET formation also disrupt other effector functions of neutrophils, the specific importance of NETs in the inflammatory context is still not clear [96]. Recent studies reported the association between NETosis dysregulation and severe autoimmune and autoinflammatory diseases [96]. It is possible that the proteolytic and oxidative processing of proteins during NETosis lead to the release of novel self-antigens, which may stimulate autoimmunity [95].

The citrullination of proteins, an important step to induce NET formation, constitutes a source of antigens that can be recognized by autoantibodies in patients with autoimmune diseases [99]. The role that citrullination plays in the pathophysiology of RA and other autoimmune diseases has been recently reviewed [73, 100]. Some patients with RA [74] and Felty's syndrome (a form of RA) [101] have autoantibodies against citrullinated histones and NETs. Anti-CCP-rich serum induces NET formation in RA patients' neutrophils [74]. The levels of citrullinated intracellular proteins correlate positively with the rate of NETosis and apoptosis in RA patients' circulating and SF neutrophils [72, 74]. Although NETosis contributes to the RA pathogenesis by generating citrullinated antigens and inducing the production of inflammatory mediators such as cytokines, chemokines, and adhesion molecules [74], a recent study has demonstrated that NETs are not a source of intracellular hypercitrullination that occurs in RA patients' synovial cells [72].

Compared with healthy individuals' neutrophils, neutrophils from RA patients with active disease exhibit increased spontaneous NETosis associated with enhanced ROS production, myeloperoxidase and elastase expression, histone-3 citrullination, and nuclear translocation of PAD-4 [102]. RA patients' SF, skin, and rheumatoid nodules contain large amounts of NETs [96], while RA patients' sera contain elevated levels of cell-free DNA, the principal components of NETs [103]. RA patients' sera and SF also induce NETosis in healthy individuals' neutrophils [102].

### 3.2. ROS-Dependent Mechanisms

The ROS production by neutrophils also participates in the pathogenesis of RA. Neutrophil activation triggers the oxidative burst that generates O_2_^•−^ via the action of NADPH oxidase, other ROS (HO^•^, ^1^O_2_, and H_2_O_2_) in subsequent reactions, and hypochlorous acid (HOCl) via the action of myeloperoxidase. NADPH oxidase is a multicomponent enzyme that is assembled at the plasma membrane during cell priming [104]. Numerous studies have indicated that (i) NADPH oxidase-derived oxygen radicals may have harmful effects in RA and (ii) RA patients' circulating neutrophils and monocytes display increased NADPH oxidase activity [89, 90, 105].

RA patients' neutrophils are primed in circulation before arriving at the inflamed synovia and they promptly respond to stimulation with soluble and insoluble ICs [19, 31]. The basal levels of total ROS, O_2_^•−^, and HO^•^, as well as the PMA- and IC-stimulated ROS generation, are significantly elevated in peripheral blood and SF neutrophils from RA patients, as compared with healthy individuals' neutrophils [26, 106]. The levels of ROS production correlate positively with DAS-28 score and the levels of C-reactive protein and anti-CCP antibodies [19, 26, 107, 108]. SF neutrophils tend to produce more O_2_^•−^ and HO^•^ and exhibit higher NADPH oxidase activity than peripheral blood neutrophils from the same individual [26]. Therefore, measuring ROS generation by peripheral blood and SF neutrophils can be an indirect indicator of inflammation and disease activity in RA patients.

Treatment of cartilage with either HOCl or H_2_O_2_ inhibits proteoglycan synthesis. The strong oxidant HOCl also degrades proteoglycans [88, 109]. Catalase and methionine mitigate the harmful effects of H_2_O_2_ and HOCl to the cartilage, respectively [88]. Proteoglycan degradation by PMA-stimulated neutrophils was unaffected by protease inhibitors that lack antielastase activity. In the same study, the H_2_O_2_-reducing agent catalase afforded significant protection against proteoglycan degradation, and antioxidants that reduced H_2_O_2_ or HOCl also decreased the activity of elastase released by PMA-activated neutrophils. These findings suggest that ROS modulate the elastase release and/or activity in neutrophil-induced cartilage degradation [110]. HOCl also mediates the activation of collagenase and gelatinase from neutrophil granules and the production of cholesterol chlorohydrins, which favors tissue injury [109].

Contrary to the traditional view that high levels of ROS mediate inflammation, some researchers argue that reduction in ROS production capacity due to polymorphisms in the respiratory burst component neutrophil cytosolic factor 1 (Ncf1 or p47^phox^) gene promotes the activation of arthritogenic T cells and leads to severe arthritis in rodents [111, 112]. Physiological ROS levels induce apoptosis of autoreactive arthritogenic T cells and prevent autoimmune responses [113]. Animals bearing Ncf1 variants associated with low burst capacity exhibit enhanced arthritis susceptibility and severity in murine models of collagen- and pristane-induced arthritis [113]. Deficient ROS production in chronic granulomatous disease patients is often associated with hyperinflammatory syndromes [114]. High levels of ROS and oxidative stress in arthritic joints may affect T-cell reactivity through different mechanisms where ROS (i) react with and change the structure of membrane and intracellular proteins involved in T-cell signaling, (ii) act as intracellular signaling molecules, (iii) affect the antigen processing and presentation, and (iv) modify the overall redox state of the extracellular milieu [111]. SF T cells also exhibit higher intracellular ROS levels than peripheral blood T cells from the same patient [115].

In addition to ROS, reactive nitrogen species (RNS) like nitric oxide and peroxynitrite participate in tissue damage in RA. ROS and RNS oxidize biomolecules, interfere in the redox balance of glutathione, increase the activation of proteolytic systems, and favor NF-*κ*B translocation to the nucleus, which in turn activates the transcription of several inflammatory mediators (see [116] for review). Therefore, ROS and RNS directly and indirectly contribute to the pathogenesis and progression of RA by promoting oxidative damage, modulating the intra- and extracellular redox status, and interfering in the activation of proteolytic enzymes and immune cells in the inflammatory environment [116].

## 4. The Use of Natural Products as Therapeutic Adjuvants to Treat RA

Patients with RA have been usually treated with nonsteroidal anti-inflammatory drugs, glucocorticoids, and disease-modifying antirheumatic drugs. Biological agents such as monoclonal antibodies and recombinant proteins that antagonize TNF-*α*, CD20, CTLA-4 (cytotoxic T-lymphocyte-associated protein 4), IL-1 receptor, and IL-6 receptor as well as therapies based on the blockade of T-cell and B-cell functions have shown efficacy to control physical signs and radiological progression in RA patients [21, 25, 117]. Most of these drugs suppress neutrophil recruitment, adherence, and functional responsiveness; such suppression usually correlates with clinical improvements in disease activity [21]. TNF-*α* and IL-6 blocking drugs also reduce serum levels of oxidative stress markers and ROS generation in peripheral blood leukocytes [118].

The aforementioned drugs are widely used in the current clinical practice, but their side effects and high costs usually limit their applicability in the chronic treatment of RA. Some RA patients like those with malignant RA do not respond to even high doses of glucocorticoids or immunosuppressive agents [21, 25]. In this sense, the use of natural products represents a promising alternative to treat rheumatic diseases, in particular by acting as therapeutic adjuvants to reduce the daily doses of conventional drugs that RA patients receive [119–121]. In the present paper, we report some recent clinical and experimental studies regarding RA treatment with plant extracts, isolated natural products, and dietary compounds, especially the studies focused on modulation of joint damage caused by neutrophil-derived ROS and granule constituents and by components of the complement system. Other relevant cellular and humoral immunological effector mechanisms that mediate the action of herbal drugs for the treatment of RA [122] and osteoarthritis [121] have been recently reviewed.

### 4.1. Clinical Studies with RA Patients

To date, several papers have reported on the efficiency of dietary supplementation with antioxidants, plant extracts, and isolated natural products to expand and support the use of such treatments in chronic inflammatory diseases [119, 120]. Other studies have suggested the necessity of therapeutic coadministration of antioxidants along with conventional drugs to treat patients with RA [119]. The hypothesis that antioxidant supplementation can be beneficial to patients with RA is reinforced by epidemiological studies that have reported that low levels of circulating antioxidants and increased oxidative stress influence the development of RA [116]. Differences among the clinical trials regarding the patients' selection criteria, disease activity, and clinical manifestations as well as the daily dose of antioxidants and duration of treatment contribute to the controversial findings on the efficacy of the use of such compounds to treat RA [119, 120]. Some recent findings are reported below and they were summarized in Table 1.

Many patients with RA and osteoarthritis around the world have used herbal preparations of* Camellia sinensis* (green tea),* Uncaria tomentosa* (cat's claw),* Tripterygium wilfordii* Hook F,* Curcuma longa* (turmeric), and* Zingiber officinale* (ginger) as therapeutic adjuvants to alleviate joint inflammation. These preparations have exhibited efficient anti-inflammatory effects in animal models of arthritis and in some clinical trials (see [126] for review).

The traditional Chinese medicine constitutes a rich source of natural compounds. The extracts of* Ganoderma lucidum* (Lingzhi) and San miao San display analgesic effects but no significant antioxidant, anti-inflammatory, or immunomodulating effects in RA patients with active disease [125]. Two Kampo medicines with synergistic effects, Jidabokuippo and Hachimijiogan, exert analgesic effects in patients with chronic arthritis who do not respond to conventional therapies [123].* Tripterygium wilfordii* Hook F extract reduced 20% of the disease activity in RA patients who were refractory to drug treatment, as compared with the placebo group [127]. The pharmacological effects of* T. wilfordii* extract are comparable to some synthetic disease-modifying antirheumatic drugs [128]. Although the dosage of the* T. wilfordii* extract taken by RA patients is considered safe and well tolerated [127], both the beneficial and the adverse effects of long-term administration of this extract alone or combined with conventional drugs should be monitored by well-designed randomized controlled trials [128].

The Mediterranean-type diet provides many antioxidant and anti-inflammatory compounds. RA patients with active disease fed with Mediterranean-type diet during twelve weeks exhibited reduced DAS-28 score and inflammatory activity and improved quality of life and physical function [129]. A group of 130 female RA patients supplemented with Mediterranean-type diet during six months displayed diminished pain scores and they increased the consumption of healthier food after the end of the study period [130]. Similarly, treatment of RA patients with capsulated rose-hip powder daily or matching placebo for 6 months improved the DAS-28 score [124].

Dietary supplementation with vitamin E (*α*-tocopherol), alone or in combination with vitamins C and A, has provided pain relief in patients with RA; this analgesic effect was independent of the anti-inflammatory effect and may complement the standard RA treatment [132]. In another study, patients with RA consumed vitamin C and margarine enriched with the antioxidants *α*-tocopherol, lycopene, palm oil carotenoids, and lutein every day for a period of 10 weeks. At the end of the study period, all laboratory measures of inflammatory activity and oxidative damage were unchanged, but the DAS-28 score and the number of swollen and painful joints were significantly reduced. The DAS-28 score increased 0.7 points after the “wash-out period” at *t* = 14 weeks; this result stresses that antioxidant supplementation improves the clinical condition of RA patients [131]. On the other hand, a group of 42 RA patients was given either *α*-tocopherol or placebo daily for 12 weeks. The patients were maintained on standard treatment with nonsteroidal anti-inflammatory drugs and disease-modifying antirheumatic drugs during the study period. Both groups reported a small but not statistically significant reduction in the Ritchie articular index and early morning stiffness [132].

### 4.2. Studies in Animal Models of RA

To study the disease pathology and examine the effect of new antiarthritic agents, scientists have widely employed animal models of human RA. Many herbal products, plant extracts, and isolated compounds diminish disease severity in the rat adjuvant- and collagen-induced arthritis models by modulating the immune response at multiple levels. These studies have been recently reviewed by Venkatesha and colleagues [122] and Nanjundaiah and colleagues [133]. We report below some other recent and relevant findings in this field (Table 2).

Decoction of the traditional Chinese herb* Oldenlandia diffusa* and its bioactive compound ferulic acid improve general health conditions, lower serum levels of IL-1*β* and TNF-*α*, and reduce redness, swelling, and hyperemia of ankle and toe joints of arthritic rats. These parameters were significantly different from the control group after 28 days of treatment [135]. The Chinese herbal formula Huo-Luo-Xiao-Ling Dan diminishes pannus formation, synovial mononuclear cell infiltration, bone and cartilage destruction, and the synovial levels of IL-18, IL-1*β*, MMP-2, and MMP-9 in arthritic Lewis rats [134].

The aqueous extract of* Withania somnifera* Dunal, a common ingredient of antiarthritic polyherbal formulations, reduces the levels of systemic oxidative stress in collagen-induced arthritis in rats: it diminishes the lipid peroxidation levels and glutathione-S-transferase activity and increases the glutathione content in plasma [137]. This plant extract diminishes the arthritic index and the production of C-reactive protein and antinuclear antibodies in an extent comparable to methotrexate [137]. The lignan eleutheroside E ((+)-syringaresinol diglucoside), the main active constituent of* Acanthopanax senticosus*, ameliorates arthritis severity in mice by inhibiting NF-*κ*B activity, inflammatory cell infiltration, pannus formation, cartilage damage, and bone erosion. This compound, which also exists in* Radix eleutherococci*, suppresses the* in vivo* production of two proinflammatory cytokines, TNF-*α* and IL-6 [141].

Administration of antioxidant compounds may have beneficial effects in RA by reducing local and systemic oxidative stress [120]. Phenolic compounds extracted from extra virgin olive oil, a widely consumed food product in Mediterranean-type diet, exert strong anti-inflammatory and antiarthritic activity in mice. These compounds reduce joint edema, cell migration, cartilage and bone erosion, and the production of proinflammatory cytokines [138].

In a mouse model of zymosan-induced acute arthritis, apocynin—a natural organic compound structurally related to vanillin that specifically inhibits NADPH oxidase activity—partially reverses the inflammation-induced inhibition of cartilage proteoglycan synthesis [139]. The antioxidant coenzyme Q_10_ potentiates the methotrexate action to reduce hind paw volume and to lower the plasma levels of the proinflammatory cytokine IL-1*α*, oxidized lipids, and proteins in adjuvant-induced arthritis in rats [140]. Coenzyme Q_10_ also mitigates the inhibitory action of methotrexate on the phagocytic capacity, oxidative burst, and metabolic activity of peripheral blood neutrophils. Hence, the coenzyme Q_10_ and methotrexate combination therapy exerts strong antiarthritic effect and balances the immunosuppression caused by methotrexate monotherapy [140].

Oral administration of the ubiquitous dietary flavonoid quercetin—a natural polyphenolic compound—to arthritic rats (150 mg/rat) clearly decreases clinical signs of the disease, as compared with untreated controls. Antiarthritic effect of quercetin correlates with decreased production of inflammatory mediators and nitric oxide by peritoneal macrophages [144]. Although quercetin does not act in synergy with vitamin E, experimental results obtained in mice fed with vitamin E-deficient diet prior to RA induction suggest that dietary deficiency of vitamin E increases inflammatory responses, and antioxidant supplementation successfully suppresses them. A significant improvement in the clinical signs of RA may require longer observation period [145]. However, quercetin does not modify plasma oxidative and inflammatory status, as well as blood pressure in patients with RA [146, 147]. Further studies are required to assess the effect of this flavonoid on oxidative stress and inflammation in humans. Other flavonoids with antiarthritic activity in animal models are 6-shogaol, naringin, hesperidin, and genistein [133].

The flavonoid hesperidin exerts marked antioxidant and antiarthritic activities in collagen-induced arthritis in rats: it lowers SF levels of the neutrophil marker elastase, nitric oxide, and lipid peroxidation; it mitigates depletion of reduced glutathione, superoxide dismutase, and catalase; it reduces paw erythema and edema and decreases cell infiltration, pannus formation, synovial hyperplasia, and bone resorption [142]. Similarly, the antiarthritic activity of* Terminalia arjuna* bark extract [136] and platycodin D [143] positively correlates with their antioxidant effect. Compared with untreated rats,* T. arjuna* extract reduces collagen-induced hind paw swelling, neutrophil infiltration, and articular elastase level (the biochemical marker of neutrophil infiltration). It also contributes to the local antioxidant defense by diminishing the levels of nitrites and oxidized lipids, attenuating the fall in reduced glutathione and superoxide dismutase levels, and increasing joint catalase activity [136]. Platycodin D, a saponin purified from* Platycodi radix*, exerts anti-inflammatory, antioxidant, and immunomodulating effects in collagen-induced arthritis in mice. This compound suppresses cytokine production in splenocytes and reduces joint swelling, the numbers of inflammatory cells infiltrated in the knee synovial cavity, and the paw levels of myeloperoxidase (a marker of neutrophil infiltration), oxidized lipids, IL-6, and TNF-*α* [143]. Together, these reports reinforce the hypothesis that modulation of free radical generation and chemical and biochemical pathways in activated neutrophils significantly contributes to reduce joint damage in arthritic animals.

### 4.3. *In Vitro* Modulation of Fc*γ*R- and CR-Mediated Effector Functions of Neutrophils

Activation of the effector functions of neutrophils via Fc*γ*Rs and CRs plays an important role in the pathogenesis of IC-mediated diseases like RA and SLE [21]. Thus, regulation of the effector potential of these receptors may be relevant to control excessive neutrophil activation at the inflammatory sites. There are few reports on the modulation of Fc*γ*R- and CR-activated neutrophil responses by natural products (Table 3).

Flavonoids have emerged as promising modulators of the neutrophil functional responses* in vitro* and* in vivo* [159, 160]. Methoxylated flavonoids isolated from* Lychnophora granmongolense* and hydroxylated flavonoids bearing the flavonol moiety—a 2,3-double bond in conjugation with a 4-oxo group and a 3-hydroxyl group—associated with the 5,7-dihydroxylation at the A-ring strongly inhibit the IC-stimulated oxidative metabolism of human and rabbit neutrophils [149–151]. The flavonols myricetin, quercetin, kaempferol, and galangin suppress the O_2_^•−^ and total ROS generation by neutrophils specifically stimulated via Fc*γ*Rs and CRs, either alone or in combination [150]. These flavonols also diminish myeloperoxidase activity and elastase release and scavenge O_2_^•−^, H_2_O_2_, hypochlorous acid, and chloramines, without affecting the NADPH oxidase activity, phagocytosis, and microbial killing capacity of neutrophils towards* Candida albicans* [151, 152, 154]. In Fc*γ*R-stimulated human neutrophils, quercetin effectively inhibits NADPH oxidase activity at concentrations higher than 20 *μ*M [153]. Quercetin and galangin suppress Fc*γ*R-stimulated ROS generation in neutrophils from RA patients with active disease who are refractory to anti-TNF-*α* drug therapy [151].

Phenylcoumarins, which are plant secondary metabolites produced by the same biosynthetic pathway of flavonoids, lower the intracellular O_2_^•−^ and total ROS levels in Fc*γ*R-stimulated neutrophils [155, 156]. These compounds effectively scavenge HOCl, inhibit myeloperoxidase activity, and slightly reduce NADPH oxidase activity [156]. The lipophilicity and electron-donating capacity markedly influence the ability of flavonols and phenylcoumarins to modulate the IC-stimulated neutrophil oxidative metabolism, myeloperoxidase activity, and free radical scavenging capacity [150, 151, 155–157].

The aqueous extract of dried ripe* Areca catechu* nuts reduces the levels of Fc*γ*Rs and CRs expression in human neutrophils and impairs the neutrophil phagocytic ability towards complement- and IgG-opsonized microspheres [148]. The combination of ascorbate (vitamin C) and tocopherol (vitamin E) inhibits ROS generation by human neutrophils stimulated via Fc*γ*R with IgG-opsonized* Staphylococcus aureus* and* Fusobacterium nucleatum* [158].

### 4.4. Modulation of the Complement System Activity and/or Activation

The complement system is a complex cascade reaction composed of nearly 50 components, activated via three main pathways, which is connected to other systems as the blood coagulation system [10, 53, 55]. Complement activation is tightly regulated to avoid host tissue damage and maintain homeostasis, and inappropriate complement activation and complement deficiency participate in the pathogenesis of various immune-mediated disorders. The exact role that the complement system plays in such diseases is not fully elucidated and more than one pathway is usually involved in disease manifestations [55, 56]. Such complexity has made the development of complement modulators a great challenge for scientists and explains, at least in part, why just few agents that regulate complement function are under clinical use (see [56, 58, 161] for review). Another challenge is to develop a selective and specific agent that blocks tissue injury without impairing the vital roles of the complement system: it helps to eliminate pathogenic microorganisms, promotes the clearance of ICs and apoptotic cells, and participates in the development of adaptive immune responses [56]. Long-term systemic suppression of complement system activity is associated with higher susceptibility to bacterial infections [55].

To date, there are few anticomplement therapeutic agents available in the clinical field to treat inflammatory diseases. The only agent approved for clinical use that directly targets the complement system is eculizumab, an anti-C5 monoclonal antibody that inhibits C5a generation and MAC formation [56, 58]. Eculizumab is widely used to treat atypical hemolytic uremic syndrome and paroxysmal nocturnal hemoglobinuria, but it has not shown much efficacy in the treatment of RA [58]. The other approved agents that target the complement system activity but also affect other systems are (i) C1 inhibitor (Serping1) concentrates, isolated from human plasma, which have been used for the treatment of sepsis and hereditary angioedema in adolescents and adults, and (ii) intravenous immunoglobulin concentrates, prepared from human plasma, which inhibit complement deposition on targets—this agent is approved for the treatment of autoimmune diseases such as Kawasaki disease and idiopathic thrombocytopenic purpura [56].

The growing recognition of the role that increased complement system activation plays in the pathogenesis of ischemic, inflammatory, and autoimmune diseases has prompted the development of novel complement-modulating agents. Some of these agents have been tested in clinical trials, such as recombinant soluble CR1, anti-C5 monoclonal antibodies, inhibitors of C3 activation, and C3aR and C5aR antagonists [56, 58, 162, 163]. The most promising candidate targets for complement inhibition in RA are (i) generation of the anaphylatoxins C3a and C5a; (ii) binding of complement fragments to complement receptors; (iii) opsonization; (iv) MAC formation and subsequent lysis of synovial tissue and SF cells [10, 58].

In this sense, there is still a great need for the discovery and development of novel agents to control excessive complement activation, which can be used in combination with the existing drugs and strategies to treat RA and other inflammatory diseases [161]. Among the natural products (Table 4), rosmarinic acid [(*R*)-*O*-(3,4-dihydroxycinnamoyl)-3-(3,4-dihydroxyphenyl)lactic acid] is one of the most promising compounds that suppress the complement system activation. This compound can be isolated from many plants like* Melissa officinalis* and* Rosmarinus officinalis*, and it can be produced in* Coleus blumei* cell cultures [164]. Rosmarinic acid inhibits the activity of the C3 convertase of the classical pathway of the complement system* in vitro* [165] and reduces prostacyclin biosynthesis without interfering in the cyclooxygenase or prostacyclin synthase activity [166].

*In vivo*, rosmarinic acid suppresses endotoxin-induced complement activation in a rabbit model of circulatory shock [166] and reduces cobra venom factor-induced paw edema and passive cutaneous anaphylaxis in rats [165]. Rosmarinic acid does not inhibit* t*-butyl hydroperoxide-induced paw edema in rats, indicating that it selectively acts on complement-dependent processes [165]. In an animal model of acute respiratory distress syndrome, administration of rosmarinic acid (10 mg/kg i.v.) prior to intravenous infusion of cobra venom factor inhibits systemic neutropenia followed by neutrophil migration to the lungs, bronchoalveolar vascular leakage, and blood pressure alterations and reduces TNF-*α* levels in serum and bronchoalveolar lavage fluid. These effects were similar to those exerted by infusion of C5aR and C3aR antagonists [169].

In two experimental models used to investigate the pathogenesis of human autosomal dominant polycystic kidney disease, conditional* Pkd1* ^−/−^ mice and Han:SPRD Cy/+ rats, the complement system inhibitor rosmarinic acid exerts beneficial effects and improves kidney function without having any severe side effects on either liver function or lipid metabolism [170]. This compound slows down the disease progression, lowers blood urea nitrogen and plasma creatinine levels, reduces the cyst index, and diminishes the complement factor B and MAC expression in renal tissues. Suppression of the complement activation decreases the infiltration of inflammatory cells, proliferation of cyst-lining epithelial cells, and renal fibrosis [170]. Rosmarinic acid also exhibits antioxidant effect, inhibits elastase activity, and suppresses the synthesis of 5-hydroxy-6,8,11,14-eicosatetraenoic acid and leukotriene B4 in human neutrophils* in vitro* [164, 165]. Together, the pharmacological properties of rosmarinic acid may be beneficial to treat RA and other inflammatory diseases and make it a promising candidate for clinical studies.

Many plant extracts and isolated compounds have been screened for their ability to inhibit the hemolytic activity of the alternative and classical pathways of the complement system* in vitro*. Interestingly, most of the bioactive compounds are glycosylated. The glycosides kaempferol 3-*O*-rhamnoside, kaempferol 3-*O*-rutinoside, and kaempferol 7-*O*-[*α*-L-rhamnopyranosyl-(1→6)]-[*β*-D-glucopyranosyl-(1→2)]-*β*-D-glucopyranoside (morindaoside) isolated from* Morinda morindoides* stem bark inhibit both pathways of complement system activation [173]. The aqueous decoction of* M. morindoides* stem bark is widely used in the African traditional medicine to treat rheumatism, hemorrhoids, and some infectious diseases [173]. Organic extracts of the flower buds of* Magnolia fargesii* and the isolated glycoside tiliroside (kaempferol 3-*O*-*β*-D-(6′′-*O*-coumaroyl)glucopyranoside) inhibit the classical pathway of the complement system more effectively than rosmarinic acid. On the other hand, the tiliroside hydrolysis products—kaempferol, astragalin, and* p*-coumaric acid—do not exert significant anticomplement activity [168]. In contrast, entadamide A and homogentisic acid, isolated from* Entada phaseoloides* seed, inhibit the hemolytic activity of the complement system more effectively than their glycosylated parent compounds entadamide A-*β*-D-glucopyranoside and homogentisic acid 2-*O*-*β*-D-glucopyranoside (phaseoloidin), respectively [172].

Four phenylethanoid glycosides—isoilicifolioside A [(*R,S*)-*β*-ethoxy-*β*-(3,4-dihydroxyphenyl)-ethyl-*O*-*α*-L-rhamnopyranosyl(1→3)-*β*-D-(6-*O*-E-caffeoyl)-glucopyranoside], ilicifolioside A [*β*-ethoxy-*β*-(3′,4′-dihydroxyphenyl)-ethyl-*O*-*α*-L-rhamnopyranosyl-(1→3)-4-*O*-caffeoyl-*β*-D-glucopyranoside], campneoside II [2-(3,4-dihydroxyphenyl)-2-hydroxyethyl-3-*O*-*α*-L-rhamnopyranosyl-4-*O*-(3,4-dihydroxycinnamoyl)-*β*-D-glucopyranoside], and isocampneoside II [(*R,S*)-7-(3,4-dihydroxyphenyl)-ethyl-*O*-*α*-L-rhamnopyranosyl(1→3)-*β*-D-(6-*O*-caffeoyl)-glucopyranoside]—isolated from* Paulownia tomentosa* wood, a plant used in the traditional Chinese medicine to treat cough, bronchitis, and asthma, exhibit strong anticomplement activity [171]. These compounds are two- and three-times more effective than tiliroside and rosmarinic acid, respectively, to inhibit the classical pathway of the complement system [171].

Podocarpaside I, which is a cycloartane-type triterpene arabinoside isolated from the roots of* Actaea podocarpa* (Ranunculaceae), exhibits moderate anticomplement activity [174]. Acetone extracts of* Bridelia ferruginea* stem bark and three isolated compounds—3,5-dicaffeoylquinic acid, 1,3,4,5-tetracaffeoylquinic acid, and the biflavanol gallocatechin-(4′-*O*-7)-epigallocatechin—strongly inhibit both the classical and alternative pathways of the complement system [167]. Two 3-methoxyflavones—quercetin 3,7,3′,4′-tetramethyl ether and myricetin 3,3′,4′,5′-tetramethyl ether—isolated from* B. ferruginea* inhibit the alternative pathway more effectively than rosmarinic acid. The quinic acid derivatives inhibit the C1 component and the terminal route of the complement system [167].

## 5. Conclusion and Future Perspectives

Many scientists have been unraveling the role that immune system cells and soluble mediators play in the pathogenesis and progression of RA, with the purpose of understanding the molecular mechanisms of this autoimmune disease, discovering new drug targets, and developing novel drugs to treat RA. Neutrophils and the complement system have emerged as relevant targets of antirheumatic and anti-inflammatory drugs. Their importance lies in the fact that these key components of the immune system directly or indirectly participate in the local and systemic manifestations of RA, as well as in the regulation of the course of inflammatory responses. Modulation of the release of cytotoxic products and proinflammatory cytokines by neutrophils and the activation of the complement system seem to be promising therapeutic strategies to reduce the disease activity and slow down the disease progression. In this sense, natural products, including herbal formulations, plant extracts, and isolated compounds, constitute a rich source of novel drugs that can intervene at multiple steps in the RA pathology. Therefore, it is vital to understand the mechanisms of action and validate the clinical efficacy of these therapies alone or combined with traditional anti-inflammatory and antirheumatic drugs.

## Figures and Tables

**Table 1:** Herbal preparations, plant extracts, and dietary antioxidants tested in clinical trials in patients with rheumatoid arthritis.

Therapy	Effect	Reference
Herbal preparation		
Jidabokuippo plus Hachimijiogan	Analgesic effect on patients with chronic arthritis who did not respond to conventional therapies.	[123]
Rose-hip	Improves general health conditions and reduces DAS-28 score in patients with RA.	[124]
San miao San	Analgesia in RA patients with active disease. No significant antioxidant, anti-inflammatory, and immunomodulating effects.	[125]

Plant extract		
*Curcuma longa *(turmeric)	Improves morning stiffness and walking time and reduces joint swelling in patients with RA.	[126]
*Ganoderma lucidum *(Lingzhi)	Analgesia in RA patients with active disease. No significant antioxidant, anti-inflammatory, and immunomodulating effects.	[125]
*Tripterygium wilfordii *Hook F	Reduces disease activity in RA patients refractory to treatment. Pharmacological effect comparable to some synthetic disease-modifying antirheumatic drugs.	[126–128]
*Uncaria tomentosa *(cats claw)	Alleviates joint pain in patients with osteoarthritis.	[126]
*Zingiber officinale* (ginger)	Reduces joint inflammation in patients with osteoarthritis.	[126]

Dietary supplementation		
Mediterranean-type diet	Reduces DAS-28 score and inflammatory activity and improves quality of life and physical function in RA patients with active disease.	[129, 130]
Vitamin C in combination with antioxidant-enriched margarine	Reduces DAS-28 score and the number of swollen and painful joints in RA patients with low disease activity. No significant anti-inflammatory and antioxidant effect.	[131]
Vitamin E	Slightly reduces the Ritchie articular index and early morning stiffness in RA patients under treatment with nonsteroidal anti-inflammatory drugs and disease-modifying antirheumatic drugs.	[132]
Vitamin E in combination with vitamins A and C	Analgesic effect in patients with RA. No significant anti-inflammatory effect.	[132]

DAS-28: disease activity score of 28 joints; RA: rheumatoid arthritis.

**Table 2:** Herbal preparations, plant extracts, isolated compounds, and dietary antioxidants tested in animal models of rheumatoid arthritis.

Therapy	Source	Model	Effect	Reference
Huo-Luo-Xiao-Ling Dan (herbal formula)		AIA rat	↓ pannus formation, ↓ synovial mononuclear cell infiltration. ↓ bone and cartilage destruction. ↓ synovial levels of IL-18, IL-1*β*, MMP-2, and MMP-9.	[134]

*Oldenlandia diffusa* (decoction)		CIA rat	Improves general health conditions. ↓ serum levels of IL-1*β* and TNF-*α*. ↓ redness, ↓ swelling, and ↓ hyperemia of ankle and toe joints.	[135]

*Terminalia arjuna* (bark extract)		CIA rat	Antioxidant effect correlates positively with antiarthritic activity. ↓ hind paw swelling, ↓ neutrophil infiltration, ↓ articular elastase level. ↓ joint levels of nitrites and oxidized lipids. Attenuates the fall in reduced glutathione and superoxide dismutase levels. ↑ joint catalase activity.	[136]

*Withania somnifera* (aqueous extract of root)		CIA rat	↓ systemic oxidative stress: ↓ lipid peroxidation levels, ↓ glutathione-S-transferase activity, ↑ glutathione content in plasma, ↑ ferric-reducing ability of plasma. ↓ arthritic index and ↓ production of C-reactive protein and antinuclear antibodies in an extent comparable to methotrexate.	[137]

Polyphenol-rich extract	Extra virgin olive oil	CIA mouse	↓ joint edema, ↓ cell migration, ↓ cartilage degradation, ↓ bone erosion. ↓ levels of proinflammatory cytokines and prostaglandin E2. ↓ expression of cyclooxygenase-2 and microsomal prostaglandin E synthase-1. ↓ translocation of NF-*κ*B to the nucleus.	[138]

Apocynin	Commercial	ZIA mouse	Partially reverses the inflammation-induced inhibition of cartilage proteoglycan synthesis.	[139]

Coenzyme Q_10_	Commercial	AIA rat	Potentiates the methotrexate action to reduce hind paw volume and to lower the levels of IL-1*α* and oxidized lipids and proteins in plasma. Mitigates the inhibitory action of methotrexate in the phagocytic capacity, oxidative burst, and metabolic activity of peripheral blood neutrophils.	[140]

Eleutheroside E	*Acanthopanax senticosus Radix eleutherococci *	CIA mouse	Ameliorates arthritis severity. ↓ NF-*κ*B activity, ↓ inflammatory cell infiltration, ↓ TNF-*α* and IL-6 production. ↓ pannus formation, ↓ cartilage damage, ↓ bone erosion.	[141]

Ferulic acid	*Oldenlandia diffusa *	CIA rat	Improves general health conditions. ↓ serum levels of IL-1*β* and TNF-*α*. ↓ redness, ↓ swelling, and ↓ hyperemia of ankle and toe joints.	[135]

Hesperidin	Commercial	CIA rat	Antiarthritic activity correlates positively with antioxidant effect. ↓ paw erythema and edema, ↓ cell infiltration. ↓ pannus formation, ↓ synovial hyperplasia, ↓ bone resorption. ↓ joint levels of neutrophil elastase, nitric oxide, and lipid peroxidation. Mitigates depletion of reduced glutathione, superoxide dismutase, and catalase.	[133, 142]

Platycodin D	*Platycodi radix *	CIA mice	Antiarthritic activity correlates positively with antioxidant effect. ↓ cytokine production by splenocytes. ↓ joint swelling and ↓ synovial infiltration of inflammatory cells. ↓ the levels of myeloperoxidase, oxidized lipids, IL-6, and TNF-*α* in the inflamed paw.	[143]

Quercetin	Commercial	AIA rat	Ameliorates clinical signs of the disease. ↓ production of inflammatory mediators and nitric oxide in macrophages.	[144]

AIA: adjuvant-induced arthritis; CIA: collagen-induced arthritis; IL: interleukin; MMP: matrix metalloproteinase; NF-*κ*B: nuclear factor-*κ*B; TNF-*α*: tumor necrosis factor-*α*; ZIA: zymosan-induced arthritis.

**Table 3:** *In vitro* effect of plant extracts, isolated compounds, and dietary antioxidants on the effector functions of neutrophils specifically stimulated via Fc*γ* and complement receptors.

Therapy	Source	Effect	Reference
*Areca catechu* (aqueous extract of dried ripe nuts)		↓ expression of Fc*γ*Rs and CRs in human neutrophils. ↓ neutrophil phagocytic ability towards complement- and IgG-opsonized microspheres.	[148]

Chrysoeriol^a^ Eriodictyol^b^ Isorhamnetin^c^ 4′-Methoxyeriodictyol^d^	*Lychnophora granmongolense *	↓ production of ROS in rabbit neutrophils stimulated via Fc*γ*Rs and/or CRs.	[149]

Quercetin (3,5,7,3′,4′-pentahydroxyflavone)	*Lychnophora ericoides *	↓ oxidative metabolism in rabbit and human neutrophils specifically stimulated via Fc*γ*Rs and CRs, either alone or in combination, without affecting the phagocytic and microbial killing capacity.	[149–151]
	Commercial	↓ human neutrophil degranulation, ↓ the activity of elastase, myeloperoxidase, and NADPH oxidase.	[151–153]
		Scavenges O_2_^•−^, H_2_O_2_, HOCl, and chloramines.	[151, 154]
		↓ Fc*γ*R-stimulated ROS generation in neutrophils from RA patients with active disease who do not respond to anti-TNF-*α* drug therapy.	[151]

Galangin (3,5,7-trihydroxyflavone)	Commercial	Strongly inhibits oxidative metabolism in rabbit and human neutrophils specifically stimulated via Fc*γ*Rs and CRs, either alone or in combination.	[150, 151]
		↓ myeloperoxidase and horseradish peroxidase activity.	[150, 151]
		↓ activity of NADPH oxidase in Fc*γ*R- and/or CR-stimulated human and rabbit neutrophils.	[153]
		Scavenges H_2_O_2_, HOCl, and chloramines.	[151]
		↓ Fc*γ*R-stimulated ROS generation in neutrophils from RA patients with active disease who are refractory to anti-TNF-*α* drug therapy.	[151]

Kaempferol (3,5,7,4′-tetrahydroxyflavone)	Commercial	↓ oxidative metabolism in rabbit and human neutrophils specifically stimulated via Fc*γ*Rs and/or CRs.	[150, 151]
		↓ NADPH oxidase activity in human and rabbit neutrophils stimulated via Fc*γ*Rs and/or CRs.	[153]
		↓ myeloperoxidase and horseradish peroxidase activity.	[150, 151]
		Scavenges H_2_O_2_, HOCl, and chloramines.	[151]

Myricetin (3,5,7,3′,4′,5′-hexahydroxyflavone)	Commercial	Slightly ↓ oxidative metabolism in rabbit and human neutrophils specifically stimulated via Fc*γ*Rs and/or CRs.	[150, 151]
		↓ human neutrophil degranulation.	[152]
		↓ activity of elastase, myeloperoxidase, NADPH oxidase, and horseradish peroxidase.	[150–153]
		Scavenges O_2_^•−^, H_2_O_2_, HOCl, and chloramines.	[151, 154]

Hydroxylated phenylcoumarins Acetoxylated phenylcoumarins	Laboratory synthesis	↓ oxidative metabolism in rabbit and human neutrophils specifically stimulated via Fc*γ*Rs.	[155, 156]
		Scavenges HOCl.	[156]
		↓ activity of myeloperoxidase, NADPH oxidase, and horseradish peroxidase.	[156, 157]

Vitamin C plus vitamin E	Commercial	↓ ROS generation by human neutrophils stimulated via Fc*γ*Rs.	[158]

CR: complement receptor; Fc*γ*R: Fc*γ* receptor; IgG: immunoglobulin G; RA: rheumatoid arthritis; ROS: reactive oxygen species; TNF-*α*: tumor necrosis factor-*α*.

^a^5, 7, 4′-trihydroxy-3′-methoxyflavone.

^b^5, 7, 3′, 4′-tetrahydroxyflavanone.

^c^3, 5, 7, 3′-tetrahydroxy-4′-methoxyflavone.

^d^5, 7, 3′-trihydroxy-4′-methoxyflavanone.

**Table 4:** Plant extracts and isolated natural products that modulate the complement system activity and/or activation.

Therapy	Source	Model	Effect	Reference
*Bridelia ferruginea* (stem bark extract)		*In vitro *	↓ classical and alternative pathways of the CS.	[167]

*Magnolia fargesii* (flower buds extract)		*In vitro *	↓ classical pathway of the CS more effectively than rosmarinic acid.	[168]

Rosmarinic acid	*Melissa officinalis* *Rosmarinus officinalis *	*In vitro *	↓ activity of the C3 convertase of the classical pathway of the CS.	[165]
		*In vivo *	↓ endotoxin-induced CS activation in a rabbit model of circulatory shock.	[166]
	Commercial	*In vivo *	↓ cobra venom factor-induced paw edema and passive cutaneous anaphylaxis in rats.	[165]
		*In vivo *	Selective inhibition of complement-dependent inflammation.	[165]
		*In vivo *	↓ cobra venom factor-induced systemic neutropenia. ↓ neutrophil migration to the lungs, ↓ bronchoalveolar vascular leakage, ↓ blood pressure alterations, ↓ TNF-*α* levels in serum and bronchoalveolar lavage fluid in an animal model of acute respiratory distress syndrome.	[169]
		*In vivo *	↓ complement factor B and MAC expression, ↓ infiltration of inflammatory cells, ↓ fibrosis in renal tissues, ↑ kidney function without impairing liver function and lipid metabolism in animal models of human autosomal dominant polycystic kidney disease.	[170]

Campneoside II^a^ Isocampneoside II^b^ Isoilicifolioside A^c^ Ilicifolioside A^d^	*Paulownia tomentosa* (wood)	*In vitro *	↓ hemolytic activity of the classical pathway of the CS more effectively than tiliroside and rosmarinic acid.	[171]

3,5-Dicaffeoylquinic acid 1,3,4,5-Tetracaffeoylquinic acid	*Bridelia ferruginea* (stem bark)	*In vitro *	↓ hemolytic activity of the classical and alternative pathways of the CS more strongly than rosmarinic acid. ↓ activation of the C1 component and the terminal route of the CS.	[167]

Entadamide A Homogentisic acid	*Entada phaseoloides* (seed)	*In vitro *	↓ hemolytic activity of the CS more strongly than their glycosylated analogues entadamide A-*β*-D-glucopyranoside and homogentisic acid 2-*O*-*β*-D-glucopyranoside (phaseoloidin), respectively.	[172]

Kaempferol 3-*O*-rhamnoside Kaempferol 3-*O*-rutinoside Morindaoside^e^	*Morinda morindoides* (stem bark)	*In vitro *	↓ hemolytic activity of the classical and alternative pathways of the CS.	[173]

Myricetin Myricetin 3,3′,4′,5′-tetramethyl ether Quercetin 3,7,3′,4′-tetramethyl ether	*Bridelia ferruginea* (stem bark)	*In vitro *	↓ hemolytic activity of the alternative pathway of the CS more effectively than rosmarinic acid.	[167]

Podocarpaside I	*Actaea podocarpa* (roots)	*In vitro *	Moderate inhibition of the hemolytic activity of the CS.	[174]

Tiliroside^f^	*Magnolia fargesii* (flower buds)	*In vitro *	↓ hemolytic activity of the classical pathway of the CS more strongly than rosmarinic acid. Its hydrolysis products kaempferol, astragalin, and *p*-coumaric acid do not exert significant anticomplement activity.	[168]

CS: complement system; MAC: membrane attack complex; TNF-*α*: tumor necrosis factor-*α*.

^a^2-(3,4-Dihydroxyphenyl)-2-hydroxyethyl-3-*O*-*α*-L-rhamnopyranosyl-4-*O*-(3,4-dihydroxycinnamoyl)-*β*-D-glucopyranoside.

^b^(*R,S*)-7-(3,4-Dihydroxyphenyl)-ethyl-*O*-*α*-L-rhamnopyranosyl(1→3)-*β*-D-(6-*O*-caffeoyl)-glucopyranoside.

^c^(*R,S*)-*β*-Ethoxy-*β*-(3,4-dihydroxyphenyl)-ethyl-*O*-*α*-L-rhamnopyranosyl(1→3)-*β*-D-(6-*O*-E-caffeoyl)-glucopyranoside.

^d^ *β*-Ethoxy-*β*-(3′,4′-dihydroxyphenyl)-ethyl-*O*-*α*-L-rhamnopyranosyl-(1→3)-4-*O*-caffeoyl-*β*-D-glucopyranoside.

^e^Kaempferol 7-*O*-[*α*-L-rhamnopyranosyl-(1→6)]-[*β*-D-glucopyranosyl-(1→2)]-*β*-D-glucopyranoside.

^f^Kaempferol 3-*O*-*β*-D-(6′′-*O*-coumaroyl)glucopyranoside.

## Abbreviations

Anti-CCP: Anti-cyclic citrullinated peptide
CR: Complement receptor
C3aR: C3a receptor
C5aR: C5a receptor
CTLA-4: Cytotoxic T-lymphocyte-associated protein 4
DAS-28: Disease activity score of 28 joints
Fc*γ*R: Fc*γ* receptor
GM-CSF: Granulocyte-macrophage colony-stimulating factor
HOCl: Hypochlorous acid
IC: Immune complex
IgG: Immunoglobulin G
IL: Interleukin
MAC: Membrane attack complex
MMP: Matrix metalloproteinase
NADPH: Reduced form of nicotinamide adenine dinucleotide phosphate
Ncf1: Neutrophil cytosolic factor 1
NET: Neutrophil extracellular trap
NETosis: Process by which neutrophils release the neutrophil extracellular traps
PAD: Peptidylarginine deiminase
PMA: Phorbol-12-myristate-13-acetate
RA: Rheumatoid arthritis
ROS: Reactive oxygen species
RNS: Reactive nitrogen species
sMAC: Soluble form of the membrane attack complex
SF: Synovial fluid
SLE: Systemic lupus erythematosus
TNF-*α*: Tumor necrosis factor-*α*.
